# Is Infant birth weight and mothers perceived birth size associated with the practice of exclusive breastfeeding in Ghana?

**DOI:** 10.1371/journal.pone.0267179

**Published:** 2022-05-05

**Authors:** Martin Wiredu Agyekum, Samuel N. A. Codjoe, Fidelia A. A. Dake, Mumuni Abu

**Affiliations:** 1 Institute for Educational Research and Innovation Studies, University of Education, Winneba, Ghana; 2 Regional Institute for Population Studies, University of Ghana, Legon, Accra, Ghana; Norwegian Institute of Public Health: Folkehelseinstituttet, NORWAY

## Abstract

**Introduction:**

Despite widespread advocacy for exclusive breastfeeding, and the associated benefits of exclusive breastfeeding for both infants and mothers, there is low prevalence in both developed and developing countries. Additionally, although several studies have been conducted on exclusive breastfeeding, very few of such studies have linked birth weight and birth size to exclusive breastfeeding. This study seeks to examine the influence of birth weight and birth size on exclusive breastfeeding.

**Methodology:**

This study adopted a sequential explanatory mixed method approach using both quantitative and qualitative methods. The quantitative approach used cross-sectional data from the 2014 Ghana Demographic and Health Survey (GDHS) and the qualitative data from interviews with exclusive breastfeeding mothers from two health facilities in La Nkwantanang Municipal Assembly in Accra, Ghana. Logistic regression analysis was used to examine whether infants birth weight and mothers perceived birth size are associated with the practice of exclusive breastfeeding while the qualitative data provided further insights into the findings from the quantitative analysis.

**Results:**

Majority (85%) of the infants in the study were of normal birth weight while 52% of the infants were perceived by their mothers to be of small birth size. The prevalence of exclusive breastfeeding was found to be 54.8%. The birth weight of infants and mothers’ perceived birth size were found to be significant predictors of exclusive breastfeeding. Infants of normal birth weight (OR = 7.532; 95% CI: 2.171–26.132) and high birth weight (OR = 6.654; 95% CI: 1.477–29.978) were more likely to be exclusively breastfed compared to low-birth-weight infants. Similarly, infants perceived to be of normal birth size were more likely (OR = 1.908; 95% CI: 1.058–3.441) to be exclusively breastfed compared to infants perceived to be of small birth size. The findings from the qualitative analysis show that birth weight rather than birth size influence mothers’ decision to practice exclusive breastfeeding.

**Conclusion:**

The findings of the study underscore the relevance of infant birth weight and perceived birth size in the practice of exclusive breastfeeding and highlights the need to incorporate both actual measurement of birth weight, and perception of infant’s birth size into policies targeted at exclusive breastfeeding. There is the need for deliberate targeted efforts at women who deliver infants of low birth weight and women who perceive their children to be of small birth size to practice exclusive breastfeeding.

## Introduction

Birth weight and birth size constitute actual and perceived measures of child anthropometrics which are important indicators for assessing the general health of infants at birth [1]. As such, infants are supposed to be weighed at birth [2] but in many developing countries, most infants are not weighed at birth due to a high proportion of deliveries occurring outside health facilities [3]. Also, not all births that occur at health facilities are recorded in the child health record book [4, 5]. However, in the absence of actual birth weight several studies using national data from developing countries, including the Demographic and Health Surveys (DHS), have reported that mothers are easily able to recall their children’s size at birth, though they may not remember the actual weight of their infants at birth [3, 6]. Consequently, birth size is often used as a proxy for birth weight and an indicator of the health of infants, though the relationship between birth weight and birth size is inconclusive owing to mixed findings in existing literature [1, 3, 6, 7].

This notwithstanding, there is empirical evidence that birth weight and birth size have consequences on early optimal feeding practices and standard growth of infants [1, 2, 8], partly because the ability of an infant to attain standard growth after birth is influenced by optimal feeding or the adequacy of dietary intake [9, 10]. The World Health Organisation recommends exclusive breastfeeding as the optimal feeding practice for infants from birth up to the first six months after birth [11]. Exclusive breastfeeding is beneficial for both the mother and the infant, and the available evidence show that the unique benefits of exclusive breast milk cannot be found in other substitutes such as commercial formulas or pasteurized donor milk [12]. Human milk, due to its bioactive compounds, has immunological, endocrinological, neuronal and psychological benefits for the child. It also delays resumption of menstruation thus serving as a family planning method for mothers [13–15]. Furthermore, evidence from research has shown that the practice of exclusive breastfeeding could avert about 13% of global infant deaths annually [16, 17]. In spite of the associated benefits of breastmilk and several global campaigns in support of exclusive breastfeeding, prevalence remains low and is even declining in many countries including Ghana [18–20]. The World Health Organisation recommends a global target of 90% prevalence of exclusive breastfeeding. Ghana however falls short of this recommended global target. Even more worrying is the decline in the prevalence of exclusive breastfeeding in Ghana from 63% in 2008 to 54% in 2014 [20].

Even though several studies have been conducted on exclusive breastfeeding globally [21–26], only a few of such studies have investigated the independent effect of birth size [27, 28] and birth weight on exclusive breastfeeding [29]. Research on the influence of both birth weight and birth size on exclusive breastfeeding is even more limited in Ghana and sub-Saharan Africa. Also, only few studies have employed both quantitative and qualitative methods in investigating the practice of exclusive breastfeeding. More importantly, no study has examined how infant birth weight and the size of a child at birth as perceived by the mother, could influence exclusive breastfeeding practice in sub-Saharan African countries such as Ghana. Consequently, a fundamental question, which is, is it the birth weight (actual measurement) or perceived size (i.e the size of a child at birth as perceived by the mother) that influences a mother decision to practice exclusive breastfeeding remains unanswered. But answering such questions will help in understanding the inhibiting and enabling factors which could help in designing and implementing effective sustainable programs aimed at targeting individuals, families, and communities to improve on optimal infant feeding behaviours, particularly exclusive breastfeeding. Against the foregoing, this study examines the influence of actual birth weight and perceived birth size on the practice of exclusive breastfeeding among infants and mothers in Ghana.

## Materials and methods

### Study design and setting

This study adopted a sequential explanatory mixed method approach using both quantitative and qualitative methods [30]. In implementing the study, secondary data from a household-based quantitative study was first analysed to examine the pattern of the responses and the quantitative findings were supported with primary qualitative data which was collected after the quantitative analysis. The quantitative arm of the study analysed nationally representative cross-sectional data from the 2014 Ghana Demographic and Health Survey (GDHS) while the qualitative arm analysed primary data from in-depth interviews with mothers (both exclusive and non-exclusive breastfeeding mothers) and health workers from two health facilities in Madina, an urban town in the Greater Accra region of Ghana.

### Sampling design

The 2014 GDHS survey used a stratified, two-stage cluster sampling design to select respondents from across the then ten (10) administrative regions of Ghana. The first stage of sampling involved the selection of clusters consisting of enumeration areas stratified by rural and urban residence [20] while the second stage involved selecting eligible respondents from households selected from a household listing of all households from clusters selected in the first stage of sampling. In all, a total of 427 clusters comprising 216 urban and 211 rural clusters were selected in the first stage of sampling while a total of 9,656 women were identified to be eligible for interview in the second stage of sampling. Of the 9,656 eligible women identified for interview, 9,396 agreed to participate in the study while 260 refused to be interviewed, yielding a response rate of 97%.

The qualitative component of the study employed purposive sampling in selecting respondents. Two health facilities which are polyclinics located in Madina, Accra were purposively selected for the study. These two polyclinics serve the population in the La Nkwantanang Municipal area. The two health facilities are well-organized polyclinics with good child welfare clinics making them ideal for the purpose of this study. Mothers who were attending child welfare clinics with their children in the two polyclinics were selected for interviewing with the help of the health workers who were staff of the polyclinics.

### Sample selection

**Fig 1:**
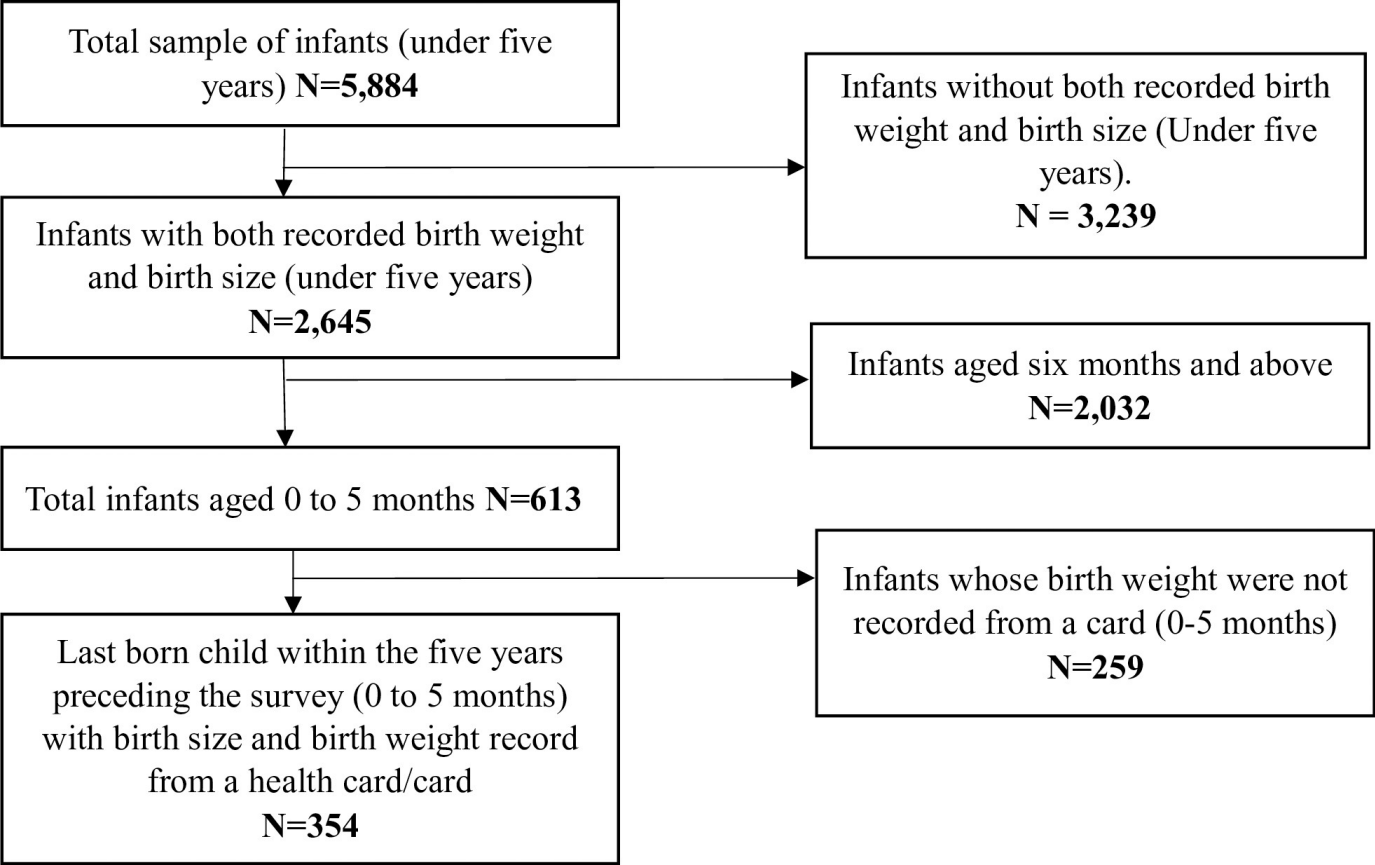
Schematic diagram showing how infants were selected from the GDHS for the study.

The unit of analysis for this study are infants who were the last-born child of their mother, born in the five years preceding the survey, and less than 6 months (0 to 5 months) old with records of both birth weight and birth size. The 2014 Ghana Demographic and Health Survey contained information on a total of 5,884 mothers with children under five years. Out of this number, about 3,239 had no records of birth weight and so were excluded from the sample for this study. The remaining 2,645 children had information about their birth size and birth weight from a child health record book or card. The sample of 2,645 children under five with both recorded actual birth weight and birth size as perceived by the mother was further limited to infants 0 to 5 months old whose total number was 613. This number was further limited to infants whose birth weight records were available from a child health record book or card and who were last born to their mothers. In all, 354 infants who had both birth size and birth weight records from a health record book or card were included in the final analytical sample (Fig 1). To ensure that the selected sample is not different from the excluded sample, a test was run to check if the background characteristics of the selected sample for the study (354) were different from the characteristics of the excluded sample (infants from 0 to 5 months with no birth weight and birth size records, n = 259). The results showed that there was no significant difference between the background characteristics of the selected sample and infants who were excluded from the study.

For the qualitative arm of the study, a total of ten exclusive breastfeeding mothers were interviewed from the two health facilities. Five exclusive breastfeeding mothers were selected from each polyclinic. Exclusive breastfeeding mothers who were selected to participate in the study met the following inclusion criteria; aged 15 to 49 years, has an infant who is less than 6 months old and practicing exclusive breastfeeding. The qualitative interviews were conducted in the preferred language of the participant and the interviews lasted about 30 minutes. The qualitative interviews were audio taped and transcribed using a professional transcriber.

### Measurement of variables

#### Dependent variable

The dependent variable for this study is exclusive breastfeeding and this was defined based on the World Health Organization’s infant feeding indicators for assessing breastfeeding practices [11]. Exclusive breastfeeding (EBF) is defined as the practice of giving infants “only breast milk from her mother or a wet nurse or expressed breast milk, and no other liquids or solids except for oral rehydration solution, or drops/syrups of vitamins or medicine [11]. For this study, exclusive breastfeeding is considered as current exclusive breastfeeding and it is based on the 24-hour recall method [27, 31]. Exclusive breastfeeding was computed from several food items categorized under breastmilk, water, liquids, milk, and solid food. Each of the food items had different types of food under each item. Infants who were fed with only breastmilk in the last 24 hours preceding the survey were coded as “1” and labeled as “Yes” for exclusive breastfeeding while those who were given foods other than breastmilk or breastmilk and other liquids, milk, water or solid food were coded as “0” and labeled as “No” for non-exclusive breastfed infants.

#### Independent variables

The main independent variables considered in this study were birth weight and birth size. During the 2014 GDHS, mothers were asked, “when (NAME) was born, was he/she very large, larger than average, average, smaller than average, or very small?” The responses were very large, larger than average, average, smaller than average and very small. For the purpose of this study, the five responses were recategorised into three (small, average and large) because the count of respondents falling within very large and very small were few. Those who responded that their babies were smaller than average and very small were recoded as “small”, average was maintained as “average”, while those who responded that their babies were larger and larger than average were recoded as “large”.

Regarding birth weight, mothers were asked if their infants were weighed at birth and if they were weighed, what was the weight of the infant in kilograms (kg) or grams (g)? Enumerators recorded the birth weight from written records or mother’s recall if such information was available. For the purposes of this study, only birth weight records from written records from the child health card/book was used as this was deemed to be more reliable than recall by the mother. The responses ranged from 1700g to 5000g and this was recategorised into three based on the WHO standard birth weight classification [32]. Infants weighing less than 2500g were categorised as “low birth weight”, infants with birth weight from 2500g to 4499g were categorised as “normal birth weight” and those with 4500g and above were categorised as “high birth weight”.

#### Control variables

The study controlled for other variables that may have an influence on exclusive breastfeeding. These variables were selected based on the reviewed literature [7, 13, 19, 21, 24, 33, 34]. The control variables included the age of the mother which was categorized as 15–19, 20–24, 25–29, 30–34, 35–39, 40–44 and 45–49. The highest level of education attained by the mother was coded as no education, primary, secondary and higher. Marital status of the mother was categorized as never married, currently married and living with partner. Mothers with children were classified based on the number of children they have ever had into mothers with one child and mothers with more than one child. Household wealth quintile was classified as poorest, poorer, middle, rich and richer. Place of residence was categorised as rural and urban residence. Ethnicity was re-categorised as Akan, Mole-Dagbani, Ewe and other ethnic groups. Religious affiliation of the mother was regrouped as Orthodox, Protestant, Pentecostal/Charismatic, other Christians, Islam and other religious groups. Employment status was re-categorised as unemployed and employed. Maternity leave by mothers was re-categorised as “no” for mothers without maternity leave after delivery and “yes” for mothers who had maternity leave after delivery. Frequency of antenatal care was measured as a continuous variable. Place of delivery was coded as public facility, private facility and other. Postnatal care was coded as “yes” for mothers who attended postnatal care and “no” for mothers who did not attend post-natal care. Mode of delivery was categorized as normal delivery and caesarean delivery. Sex of child was classified as males and females. Birth order of infants was measured as a continuous variable and age of child was analysed as a categorical variable and classified as 0, 1, 2, 3, 4 and 5 months.

### Ethics statement

The study protocol for the GDHS was approved by the Ghana Health Service Ethical Review Committee and the Institutional Review Board of ICF International. Informed consent for voluntary participation was obtained from participants before being interviewed.

Ethical clearance for the qualitative study was obtained from the Ethics Review Committee of Ghana Health Service (GHS-ERC #005/08/2017) and the University of Ghana Ethics Committee for the Humanities (ERC #020/17-18). Informed consent was obtained from each participant before they were interviewed. Additionally, the purpose of the study including the general objectives, benefits, and risk of taking part in the study were explained to the participants. The participants were informed about strict confidentiality and anonymity of their responses. They were also informed about their right to stop participating in the study at any point if they desired to do so. Those who agreed to be part of the study signed an informed consent form and, if they could not read or write, they provided a thumb print to participate in the study.

### Method of analysis

The quantitative data were anaylsed using IBM SPSS Statistics for Windows (SPSS) Version 25. The characteristics of the study sample were described using descriptive statistics. Cross tabulations and chi square test were used to test for the associations between the dependent variable and other variables (independent and control variables). Logistic regression analysis was used to examine the influence of birth weight and birth size on exclusive breastfeeding. The qualitative data were analyzed based on emerging themes from the data. Atlas.Ti was used to analyse the data. Deductive coding based on the literature review and inductive coding (data-driven) were employed in analysing the qualitative data. The first author read through all the transcripts to identify the deductive codes. The codes were validated by the other authors after they had read through the transcripts. The initial stage of the analyses involved reading the transcripts repeatedly to gain familiarity with the text as well as noting initial codes. This was followed by assigning codes to the relevant segments of the transcript. At this stage, different codes from the transcripts were sorted into potential themes, as all relevant codes were collated within the identified themes [35]. Similar codes were put together to form a theme and these were grouped at three levels: basic, organising and global themes.

## Results

### Exclusive breastfeeding, birth weight, birth size and characteristics of mothers and infants

Table 1 presents the characteristics of mothers and infants from the quantitative data. The results show that a little more than half of the infants (54.8%) were exclusively breastfed while about 45.2% were not exclusively breastfed within the 24 hours preceding the survey. In terms of birth weight, majority (85.0%) of the infants had normal birth weight while a small proportion (6.5%) had low birth weight. With regards to birth size, about 52.0% of the infants were perceived by their mothers to be of small birth size, while a little lower than one third (32.2%) were perceived to be of normal birth size and 15.8% were perceived to be of large birth size.

**Table 1:** Background characteristics of mothers and infants in the study (N = 354).

	% (N)	Exclusive breastfeeding		
		Non-EBF % (N)	EBF % (N)	P value
**Exclusive breastfeeding**				
Yes	54.8 (194)			
No	45.2 (160			
**Birth Weight**				
Low	6.5 (23)	78.3 (18)	21.7 (5)	
Normal	85.0 (301)	42.9 (129)	57.1 (172)	0.004**
High	8.5 (30)	43.3 (13)	56.7 (17)	
**Birth Size**				
Small	52.0 (184)	46.7 (86)	53.3 (98)	
Normal	32.2 (114)	37.7 (43)	62.3 (71)	0.079
Large	15.8 (56)	55.4 (31)	44.6 (25)	
**Age of mother**				
15–19	6.8 (24)	58.3 (14)	41.7 (10)	
20–24	21.2 (75)	49.3 (37)	50.7 (38)	
25–29	26.3 (93)	47.3 (44)	52.7 (49)	0.369
30–34	22.0 (78)	38.5 (30)	61.5 (48)	
35–39	17.5 (62)	41.9 (26)	58.1 (36)	
40–44	5.1 (18)	33.3 (6)	66.7 (12)	
45–49	1.1 (4)	75.0 (3)	25.0 (1)	
**Educational level**				
No education	29.1 (103)	45.6 (47)	54.4 (56)	
Primary	19.5 (69)	49.3 (34)	50.7 (35)	
Junior High School	34.5 (122)	42.6 (52)	57.4 (70)	0.858
Secondary	10.4 (37)	48.6 (18)	51.4 (19)	
Higher	6.5 (23)	39.1 (9)	60.9 (14)	
**Marital status**				
Never married	10.2 (36)	55.6 (20)	44.4 (16)	
Married	68.9 (244)	41.4 (101)	58.6 (143)	0.097
Living with Partner	20.9 (74)	52.7 (39)	47.3 (35)	
**Children ever born**				
Mothers with one child	22.9 (81)	49.4 (40)	50.6 (41)	
Mothers with two or more children	77.1 (273)	44.0 (120)	56.0 (153)	0.389
**Employment status**				
Not working	35.3 (125)	47.2 (59)	52.8 (66)	
Working	64.7 (229)	44.1 (101)	55.9 (128)	0.576
**Ethnicity**				
Akan	34.5 (122)	55.7 (68)	44.3 (54)	
Ewe	10.2 (36)	36.1 (13)	63.9 (23)	0.015*
Mole-Dagbani	34.7 (123)	36.6 (45)	63.4 (78)	
Other	20.6 (73)	46.6 (34)	53.4 (39)	
**Place of residence**				
Urban	49.2 (174)	48.9 (85)	51.1 (89)	
Rural	50.8 (180)	41.7 (75)	58.3 (105)	0.175
**Maternity leave by mother**				
No	88.4 (313)	46.6 (146)	53.4 (167)	
Yes	11.6 (41)	34.2 (14)	65.8 (27)	0.131
**Religious group**				
Orthodox	18.4 (65)	27.7 (18)	72.3 (47)	
Protestants	9.6 (34)	52.9 (18)	47.1 (16)	
Pentecostal/Charismatic	31.1 (110)	48.2 (53)	51.8 (57)	0.072
Other Christians	12.4 (44)	50.0 (22)	50.0 (22)	
Muslims	23.2 (82)	48.8 (40)	51.2 (42)	
Other religious groups	5.4 (19)	47.4 (9)	52.6 (10)	
**Wealth quintile**				
Poorest	29.3 (104)	31.7 (33)	68.3 (71)	
Poorer	14.7 (52)	57.7 (30)	42.3 (22)	
Middle	19.8 (70)	52.9 (37)	47.1 (33)	0.013*
Richer	20.1 (71)	46.5 (33)	53.5 (38)	
Richest	16.1 (57)	47.4 (27)	52.6 (30)	
**Place of delivery**				
Home	9.9 (35)	57.1 (20)	42.9 (15)	
Government Facility	81.1 (287)	43.6 (125)	56.4 (162)	0.306
Private Facility	9.0 (32)	46.9 (15)	53.1 (17)	
**Postnatal within two months**				
No	26.0 (92)	45.6 (42)	54.4 (50)	
Yes	74.0 (262)	45.0 (118)	55.0 (144)	0.919
**Mode of delivery**				
Caesarean	13.6 (48)	39.6 (19)	60.4 (29)	
Normal delivery	86.4 (306)	46.1 (141)	53.9 (165)	0.401
**Sex of child**				
Male	52.5 (186)	46.2 (86)	53.8 (100)	
Female	47.5 (168)	44.0 (74)	56.0 (94)	0.679
**Age of Child (months)**				
0	4.2 (15)	20.0 (3)	80.0 (12)	
1	13.6 (48)	27.1 (13)	72.9 (35)	
2	18.9 (67)	29.8 (20)	70.2 (47)	0.000***
3	20.3 (72)	45.8 (33)	54.2 (39)	
4	18.4 (65)	53.9 (35)	46.1 (30)	
5	24.6 (87)	64.4 (56)	35.6 (31)	
**Continuous variables**	**Minimum, Maximum (Mean, ± SD)**	**Correlation coefficient**		
Antenatal care	0, 20 (6.4, 2.8)	0.054		0.314
Birth order	1.0, 9.0 (3.1, 1.9)	-0.045		0.401

Source: GDHS, 2014 N = 354

*p<0.05

**p<0.01

***p<0.001.

According to the results presented in Table 1, slightly more than a quarter (26.3%) of the mothers were 25–29 years old, about one-third (34.46%) had attained Junior High School level of education and only 6.5% had attained higher level education. In terms of marital status, more than two-thirds of the mothers (68.9%) were married. Slightly more than a quarter (29.3%) of the mothers belonged to households in the poorest wealth category, while one-fifth (20.1%) belonged to households in the richer quintile. With regards to ethnicity, about one third of the mothers were identified as Mole-Dagbani (34.7%) and another one-third as Akan (34.5%). In terms of religion, about one third (31.1%) of the mothers were affiliated with the Pentecostal/Charismatic faith.

The number of times mothers’ made an ANC visit when they were pregnant with the last born child ranged from 0 to 20 times with an average of 6.4 visits. Generally, more than three-quarters of the mothers had their last child delivered at government health facilities and majority of the mothers attended postnatal care within two weeks after delivery. With regards to the mode of delivery, 86.4% of the mothers had normal delivery whilst 13.6% had cesarean delivery. Also, about 88.4% of the mothers did not take maternity leave after delivery. Regarding the infants, almost a quarter (24.6%) were five months old.

For the qualitative study, ten (10) exclusive breastfeeding mothers were interviewed. The age range for the participants was 25 to 40 years. About six (6) of the participants had Junior High School education, two (2) had tertiary education and one (1) had primary education. With regards to marital status, eight (8) of the participants were married while two (2) were not married. Also, seven (7) participants were Christians while three (3) were Muslims.

The quantitative results further show that birth weight, ethnicity, wealth quintile and age of infants were associated with exclusive breastfeeding (Table 1). With regards to birth weight, about 57.1% of normal birth weight infants were exclusively breastfed while 56.7% of high birth weight infants were exclusively breastfed. In contrast, 78.3% of low-birth-weight infants were not exclusively breastfed. In terms of wealth, more than two-thirds (68.3%) of infants from poorest households were exclusively breastfed, while slightly more than half (53.5%) of infants from richer households were exclusively breastfed. The lowest proportion (42.3%) of infants from the various household wealth quintile categories who exclusively breastfed was observed among the poor category (Table 1). Among the various ethnic groups, except for infants belonging to Akan ethnic group (44.3%), all the other ethnic groups had more than half of their infants being exclusively breastfed and of all the ethnic groups, Ewe women had recorded the highest proportion of exclusively breastfed infants. In terms of age of child and exclusive breastfeeding, the proportion of infants exclusively breastfed reduced with increase in child’s age. About four-fifth (80.0%) of infants who were less than one month were exclusively breastfeed while slightly more than one-third (35.6%) of infants who were five months old were exclusively breastfed.

### Factors affecting exclusive breastfeeding among infants who were 0–5 months old surveyed in this study

The results of the binary logistic regression in Table 2 show the factors that predict exclusive breastfeeding in Ghana. In Model 1, birth size was not associated with exclusive breastfeeding. Model 2 shows that birth weight was associated with exclusive breastfeeding. In Model 3 both birth weight and birth size were associated with the practice of exclusive breastfeeding. Model 4, shows that birth weight, birth size, household wealth quintile, ethnicity, religion and infant age were associated with exclusive breastfeeding. Compared to low-birth-weight infants, infants who were of normal (OR = 7.532; 95% CI: 2.171–26.132) and high birth weight (OR = 6.654; 95% CI: 1.477–29.978) were more likely to be exclusively breastfed (Model 4). The results in Model 4 further shows that infants perceived to be of normal birth size were more likely (OR = 1.908; 95% CI: 1.058–3.441) to be exclusively breastfed compared to infants perceived to be of small birth size. The results from the quantitative data also show that both birth weight and perceived birth size were associated with exclusive breastfeeding behavior. However, from the qualitative results, majority of the mothers who were interviewed mentioned that they were influenced by the birth weight of their infants to practice exclusive breastfeeding. These were mostly mothers with normal and high birth weight infants. For instance, some mothers indicated that birth weight is important to them than the perceived birth size of infants and that influenced their decision to practice exclusive breastfeeding. One of the mothers said that;

*“When I give him other food*, *he will become plump and not heavy but when you breastfeed*, *he will gain the normal weight and he won’t fall sick so I considered the birth weight to practice exclusive breastfeeding”* (Exclusive breastfeeding mother, 33 years, not married).

*“The birth weight was very important in my decision to practice exclusive breastfeeding”* (Exclusive breastfeeding mother 32 years, married).

**Table 2:** Binary logistic regression showing predictors of exclusive breastfeeding among infants 0–5 months old surveyed in this study.

Variables	Model 1	Model 2	Model 3	Model 4 Full model
	Birth size	Birth weight	Birth weight and birth size	
	OR (CI 95%)	OR (CI 95%)	OR (CI 95%)	OR (CI 95%)
**Birth size [Small]**				
Normal	1.449 (0.900–2.334)		1.669 (1.019–2.733) *	1.908 (1.058–3.441) *
Large	0.708 (0.388–1.291)		0.856 (0.458–1.602)	0.889 (0.421–1.881)
**Birth weight [Low]**				
Normal		4.800 (1.736–13.268) **	5.166 (1.818–14.677) **	7.532 (2.171–26.132) **
High		4.708 (1.381–16.042) *	5.506 (1.575–19.246) **	6.654 (1.477–29.978) *
**Age of mother [15–24]**				
25–34				1.563 (0.741–3.295)
35+				1.715 (0.637–4.622)
**Education of mother [No education]**				
Primary				1.139 (0.516–2.515)
Junior High School				1.892 (0.845–4.234)
Secondary				2.777 (0.911–8.458)
Higher				1.557 (0.375–6.462)
**Marital status [Never married]**				
Married				2.090 (0.777–5.618)
Living with Partner				1.055 (0.382–2.917)
**Children Ever Born [One]**				
2+				1.171 (0.486–2.821)
**Household Wealth quintile [Poorest]**				
Poorer				0.280 (0.118–0.665) **
Middle				0.455 (0.184–1.103)
Richer				0.463 (0.169–1.271)
Richest				0.395 (0.119–1.307)
**Employment status of the mother [Not working]**				
Working				1.092 (0.615–1.940)
**Ethnicity [Akan]**				
Ewe				3.629 (1.392–9.464) *
Mole-Dagbani				1.808 (0.774–4.222)
Other				1.890 (0.816–4.377)
**Place of residence [Urban]**				
Rural				1.312 (0.648–2.659)
**Maternity leave [No]**				
Yes				1.203 (0.484–2.989)
**Religion [Orthodox]**				
Protestant				0.509 (0.169–1.533)
Pentecostal/Charismatic				0.558 (0.240–1.297)
Other Christians				0.413 (0.147–1.159)
Islam				0.384 (0.156–0.944) *
Other religious groups				0.904 (0.245–3.338)
**Antenatal Care**				0.933 (0.843–1.033)
**Place of delivery [Home]**				
Government facility				1.990 (0.803–4.932)
Private facility				2.221 (0.630–7.830)
**Postnatal [No]**				
Yes				0.905 (0.489–1.673)
**Mode of delivery [Caesarean]**				
Normal delivery				1.375 (0.614–3.080)
**Sex of child [Male]**				
Female				1.259 (0.734–2.160)
**Age of child [< 1 month]**				
1 month				0.607 (0.126–2.917)
2 months				0.458 (0.097–2.158)
3 months				0.288 (0.064–1.298)
4 months				0.143 (0.030–0.667) *
5 months				0.082 (0.018–0.371)**
**Birth order number**				0.971 (0.782–1.207)
***Goodness of fit***				
*Nagelkerke R* ^*2*^	*0*.*0105 (1*.*05%)*	*0*.*0230 (2*.*30%)*	*0*.*0344 (3*.*44%)*	*0*.*2021 (20*.*21%)*
*Number of respondents*	*354*	*354*	*354*	*354*

Source: GDHS, 2014

*p<0.05

**p<0.01

***p<0.001 [] Reference category.

Another mother was of the view that weight helps her to monitor the growth of the infants and this was very important in her decision to practice exclusive breastfeeding than birth size. She mentioned that;

*“Because the birth weight helps you to know that the child is growing*. *It is always important than the size*, *so I considered the birth weight of the child to practice exclusive breastfeeding*” (Exclusive breastfeeding mother, 36 years, married).

A few of the mothers who were influenced by birth size to practice exclusive breastfeeding also indicated that birth size helps them to easily know the changes in the size of infants than the birth weight. One woman indicated that;

***“****I don’t know anything about the weight*. *I was influenced by the birth size to practice exclusive breastfeeding”* (Exclusive breastfeeding mother, 33 years, married).

Also, another mother indicated that birth size helps to see how large or small the child has grown at any time. She reported that;

***“****The size will help me to see how big he or she is*. *I would want to maintain it and don’t want her to be falling sick*. *I won’t introduce any other thing*. *I will maintain it or increase it”* (Exclusive breastfeeding mother, 40 years, married).

Other factors that were found to influence exclusive breastfeeding in the quantitative analysis were household wealth quintile, ethnicity, religion and the age of the infant. The results indicate that mothers from the Ewe ethnic group were more likely (OR = 3.629; 95% CI: 1.392–9.464) to be practicing exclusive breastfeeding compared to mothers from the Akan ethnic group. In terms of religious affiliation, Muslim mothers were 0.384 times as likely to be practicing exclusive breastfeeding compared to mothers from the orthodox faith (OR = 0.384; 95% CI: 0.156–0.944). Regarding wealth status, mothers from poorer households were less likely (OR = 0.280; 95% CI: 0.118–0.665) to be practicing exclusive breastfeeding compared to mothers from the poorest household wealth quintile. Similar findings were observed in the qualitative results, where a mother reported that;

*“If I give him the canned food and I don’t have money to buy more then it means he will starve so I will rather practice exclusive breastfeeding”* (Exclusive breastfeeding mother, 32 years, married)

The results further show that there is an inverse relationship between age of infants and exclusive breastfeeding. Infants who were four months old, had a lower likelihood (OR = 0.143; 95% CI: 0.030–0.667) of being exclusively breastfed compared to infants who were less than a month old. Also, infants who were five months old, had a lower likelihood (OR = 0.082; 95% CI: 0.018–0.371) of being exclusively breastfed compared to infants who were less than a month old. The findings from the qualitative analysis complements the findings from the quantitative data. The results revealed that infants are given water and food before the sixth month because mothers expressed that at that age breastmilk alone is not enough for infants when they are growing. Also, mothers are encouraged by their biological mothers and in-laws to give infants water before their sixth month.

## Discussion

This study examined the influence of infants’ birth weight and perceived birth size on exclusive breastfeeding in Ghana. The prevalence of exclusive breastfeeding among infants less than six months per the 24-hour recall method was 54.8%. This implies that slightly more than half of the infants were exclusively breastfed during the 24 hours preceding the survey. This prevalence is below the 90% prevalence recommended by the World Health Organization [36]. This, therefore, underscores the need to improve on infant feeding practice by adopting strategies to encourage exclusive breastfeeding. Further, the prevalence of exclusive breastfeeding as was found in this study (54.8%) is lower than the 63% prevalence recorded in the 2008 Ghana Demographic and Health Survey. This suggests that the prevalence of exclusive breastfeeding in Ghana is on the decline. The probable reason for the decline could be attributed to inadequate knowledge about exclusive breastfeeding leading to misconceptions about exclusive breastfeeding coupled with family influence and low breast milk production by mothers [25, 37]. In addition, some mothers worry that their children will not eat well especially after six months of exclusive breastfeeding when they are introduced to solid food [25]. This deters mothers from practicing exclusive breastfeeding thereby leading to the introduction of foods other than breastmilk to infants at an early age. This is concerning because the practice of non-exclusive breastfeeding could expose infants to morbidity and mortality and this could have an effect on neonatal and infant mortality in Ghana.

The findings also indicate that birth weight is associated with exclusive breastfeeding and is consistent with findings from other studies [15, 29, 38–40] that have also found that birth weight significantly predicts exclusive breastfeeding practice. In this current study, the odds of mothers practicing exclusive breastfeeding for infants with normal or high birth weight was higher than infants with low birth weight. The probable reason could be that in recent times, there has been an increase in advertisement of formula foods in Ghana. As a result of this, friends and family members recommend formula foods to mothers of low birth weight infants with the perception that exclusive breastfeeding alone cannot help such infants put on weight [23]. In addition, mothers-in-law or grandmothers encourage mothers with low birth weight infants to introduce other foods to infants [41]. Sometimes low birth weight infants are kept for longer periods at the intensive care unit due to their physiological characteristics which therefore reduces contact with their mothers. Additionally, low birth weight infants sometimes find it difficult initiating and maintaining breastfeeding [15, 38]. This limits the ability of mothers to exclusively breastfeed their low-birth-weight infants.

Also, the results show that perceived birth size was associated with exclusive breastfeeding practice. Normal birth size infants were more likely to be exclusively breastfed compared to small birth size infants. Similar findings have been reported in sub-Saharan Africa [27, 28]. This finding is plausible because the decision of the mother to exclusively breastfeed her normal birth size infant could be influenced by the perception of the mother. Perception is influenced by factors such as cultural, environmental and individual experiences [1]. These factors influence the decision of mothers to perceive the size of their infants as small, normal or large at birth. Evidence has shown that perception changes and prior to the twentieth century, society valued large body size but recently, there is a value for normal body size [42]. There is also recognition that in Ghana and other sub-Saharan Africa countries, people now value normal birth size and this influence feeding practices of infants. The decision of the mother to practice exclusive breastfeeding, therefore, depends on the satisfaction of the perceived birth size of the infant [42]. This probably explains why mothers who perceive the size of their infants to be normal were more likely to be exclusively breastfed compared to small birth size infants.

The qualitative findings of the present study also showed that though mothers agreed that they consider both perceived birth size and birth weight in their decision to practice exclusive breastfeeding, most of these mothers mentioned that birth weight influences their decision to practice exclusive breastfeeding. This underscores the relevance of actual measurement rather than perception in decision making. The plausible reason could be that mothers are educated on the weight of infants at both antenatal and postnatal clinics. In addition, infants’ weight is taken at birth and at every child welfare clinic and this emphasizes the relevance of birth weight rather than birth size. The ability to scientifically monitor or objectively weigh infants could have influenced mothers to consider the birth weight of the infant rather than the perceived birth size to practice exclusive breastfeeding. Additionally, the qualitative study was done in an urban environment where most mothers are literate and could read from the birth record book. Their ability to read from the book and understand the relevance of birth weight could also influence their decision to rely on the birth weight rather than the birth size. Furthermore, mothers indicated that breastmilk helps infants to put on weight compared to formula foods. In their view, formula foods make infants large in size but does not allow for an increase in weight. Hence, the interest of mothers to have children with good weight rather than large size motivated them to consider birth weight as an important factor in exclusive breastfeeding.

In addition, the results from both the quantitative and qualitative data explain the nuances of wealth being a significant factor in exclusive breastfeeding. The pattern shows that mothers from the poorest household were more likely to practice exclusive breastfeeding. The results from the present study corroborates the observed patterns in other studies [31, 43]. The practice of exclusive breastfeeding has economic benefit as mothers do not spend money on preparing food for infants when practicing exclusive breastfeeding. This economic benefit probably influenced mothers from the poorest household to practice exclusive breastfeeding as their income level is low.

Furthermore, the results of the present study showed that ethnicity is associated with exclusive breastfeeding and women from the Ewe ethnic group, predominantly from the Volta region of Ghana had higher odds of their infants being exclusively breastfed as compared to Akan women. The results is similar to findings from other settings in Ghana [24, 44, 45]. Some ethnic groups have cultural and traditional beliefs that facilitate and improve exclusive breastfeeding practice [44]. Among the Ewes, there is support from kin group and friends for nursing mothers which reduces the household burden and allows them to have more time for the infants [46]. Also, early introduction of Community-Based Health Planning and Services (CHPS) in the Volta region could have helped promote exclusive breastfeeding practice among the Ewes. In Ghana, Adongo *et al*., [47] found that policies such as Community-Based Health Planning and Services (CHPS) improve health education on nutrition and exclusive breastfeeding. Mothers are educated on nutrition, such as the required nutrients that help to build strong immunity for children. The introduction and piloting of Community-Based Health Planning and Services (CHPS) in the Nkwanta District in the Volta region which assisted in providing primary health care could have also influenced exclusive breastfeeding among Ewe women. Volta region was part of the piloting regions after the success in Navrongo in the Upper East region. The program involved community members and health service personnel in providing primary health care. In addition, community engagement such as durbars and provision of service at home were practiced. Studies have shown that the Nkwanta project in the Volta Region aided in improving health care utilization [48]. The ripple effect could probably explain the reasons why infants born to Ewe mothers were found to have been exclusively breastfed compared to infants of mothers of other ethnic groups.

Also, age of the child was found to significantly predict exclusive breastfeeding. The results show that there is an inverse relationship between the age of the child in months and exclusive breastfeeding. Similar findings have been reported in developing countries and Ghana [23, 49, 50]. In Ghana, Diji *et al*., [23] reported that exclusive breastfeeding reduces as child’s age increases. The plausible reason could be that at an early age of the child, mothers may feel comfortable practicing exclusive breastfeeding but as the age of the child increases, mothers may introduce other supplementary food with the notion that breast milk alone is not enough.

In addition, practices such as post-partum and maternity leave could influence the practice of exclusive breastfeeding. In Ghana, women are given three months of maternity leave thus creating the opportunity to exclusively breastfeed their children in that period. But exclusive breastfeeding becomes difficult for mothers when they resume work after the three months maternity leave. This could explain why exclusive breastfeeding practice is inversely associated with infants’ age. The qualitative results revealed that maternity leave influences exclusive breastfeeding behavior. Some participants mentioned that they are affected by the three months maternity leave hence they take leave of absence after their maternity leave in order to complete six months exclusive breastfeeding.

In spite of these findings, the study has some limitations that are worth mentioning. Firstly, the 24-hour recall method used for computing exclusive breastfeeding was obtained from mothers who were still breastfeeding their 0–5 months old infants. This method measures the “current status” of exclusive breastfeeding but not completed exclusive breastfeeding. Despite these limitations, the 24-hour recalled method is recommended by the World Health Organisation to measure exclusive breastfeeding of infants less than 6 months. Also, the subjective measure of birth size is affected by recall bias. Mothers may forget the size of the infant at birth and may recall incorrectly at the time of the survey. Hence, infants who are the last-born child of their mothers were used in this study to reduce the recall bias of how mothers perceived the birth size of their infants. In spite of these limitations, a mixed method approach provided a more nuanced explanation on the influence of infant birth weight (actual measurement) and mother’s perceived birth size on the practice of exclusive breastfeeding practice in Ghana. This, therefore, serves as a strength for the study as most studies on exclusive breastfeeding in Ghana and SSA are either quantitative or qualitative.

## Conclusions

The results show that birth weight and birth size were associated with exclusive breastfeeding among infants 0 to 5 months in Ghana. While exclusive breastfeeding is recommended for every newborn baby, women in Ghana tend to base their decision to exclusive breastfeeding on the birth weight of the infant and or their perception of the size of their baby at birth. Advocacy messages should dispel this misconception and restate the place of exclusive breastfeeding as relevant for every baby regardless of their birth weight or perceived size at birth. Furthermore, other factors in addition to birth weight and the perceived size of infants, for example the ethnicity and religion of mothers and the age of the infant as well as household wealth predict the practice of exclusive breastfeeding in Ghana. Policy interventions on exclusive breastfeeding must there consider these other factors in addition to infant birth weight and birth size as perceived by mothers. Also, health workers need to be sensitized to record the birth weight of infants after delivery as well as encourage mothers to keep their health record books. Efforts should also be targeted at women with low-birth-weight infants and mothers who perceive their infants to have small birth size to practice exclusive breastfeeding. In addition, programs aimed at the promotion of exclusive breastfeeding should incorporate ethnic and religious differences as well as targeting women belonging to poor households to encourage them to continue practicing exclusive breastfeeding.

## Supporting information

S1 Appendix Interview guide. (DOCX)

S2 Appendix Quantitative data. (XLSX)

S3 Appendix Qualitative data. (DOCX)
