# Supplementary material for: Is Infant birth weight and mothers perceived birth size associated with the practice of exclusive breastfeeding in Ghana?
Source: PLoS One. 2022 May 5;17(5):e0267179. doi: 10.1371/journal.pone.0267179 (PMC9070922; doi:10.1371/journal.pone.0267179)
Supplement: S1 Appendix — (DOCX) [file pone.0267179.s001.docx]

**Appendix A**

**In-depth Interview guide**

**Exclusive breastfeeding mothers_**

**Section 1: Demographics**

- 1. What is your highest level of education?
  2. What is your ethnic affiliation?
  3. What is your religious affiliation?

Specify your denomination if a Christian?

- 1. What is your marital status?
  2. What is your employment status? If yes, indicate what you do?
  3. How many children do you have?
  4. How old are you?
  5. What is your age when you first gave birth or age for your last child?
  6. Locality of residence?
  7. Sex of child?
  8. Mode of delivery?
  9. How old is your child?

**Section 2: Child characteristics**

2.1 What was the birth size of your child when he/she was born?

2.2 Were you satisfied or not satisfied with the size of your child when he/she was born?

Probe: Reason for been satisfied or not satisfied

2.3 What was the birth weight of your child? (Check weighing card book to record)

2.4 Were you informed about the birth weight of the child after delivery?

2.5 Did you consider the birth size or birth weight to be more important to you?

**Section 3: Decision to exclusively breastfeed**

- 1. Did you plan to exclusively breastfeed your child after delivery?

Probe: Reason for intention to exclusively breastfeed

3.2 What informed your decision to practice exclusive breastfeeding?

Probe: Birth weight and birth size

Probe: Were you told at the hospital to practice exclusive breastfeeding? if yes, by who?

Probe: Were you taken through nutrition lessons during antenatal clinics and after delivery

breastfeeding management? If yes, what lessons

Probe: Partner support, in-law support, economic reasons

**Section 4: Management/Practice of exclusive breastfeeding**

4.1 What has been the most difficult part of practicing exclusive breastfeeding?

4.2 What has been the most satisfying part of practicing exclusive breastfeeding?

4.3 How have you managed your experience?

Probe: support from family members, friends, etc

Influence of ethnicity, wealth, religion, birth weight and birth size on exclusive breastfeeding?

**Section 5: Benefits of exclusive breastfeeding**

- 1. What are the benefits of practicing exclusive breastfeeding?

Probe: Benefit for the child.

Benefit for the mother

**Section 6: Challenges of exclusive breastfeeding**

6.1 What are the challenges you faced in practicing exclusive breastfeeding?

Probe: Challenges from the child

Challenges from the mother

Social, religion, work, environment, family, friends

6.2 Are there cultural issues promoting the practice of exclusive breastfeeding?

Probe: for local beliefs

6.3Are there cultural issues inhibiting the practice of exclusive breastfeeding?

Probe: for local beliefs

- 1. How are you coping?
