# Supplementary material for: Is Infant birth weight and mothers perceived birth size associated with the practice of exclusive breastfeeding in Ghana?
Source: PLoS One. 2022 May 5;17(5):e0267179. doi: 10.1371/journal.pone.0267179 (PMC9070922; doi:10.1371/journal.pone.0267179)
Supplement: S3 Appendix — (DOCX) [file pone.0267179.s003.docx]

**Themes and their sample quotes on exclusive breastfeeding**

| **Global Themes** | **Organizing theme** | **Basic theme** | **Sub-basic theme** | **Quotes** |
| --- | --- | --- | --- | --- |
| Experiences of mothers and health workers | Considering birth weight or birth size to practice EBF | Birth weight |  | *“Because the birth weight helps you to know that the child is growing. It is always more important than the size, so I considered the birth weight of the child to practice exclusive breastfeeding*” (P9, Exclusive breastfeeding mother).  *“When I give him other food, he will become plump and not heavy but when you breastfeed, he will gain the normal weight and he won’t fall sick so I considered the birth weight to practice exclusive breastfeeding”* (P4, Exclusive breastfeeding mother).  *“The birth weight was very important in my decision to practice exclusive breastfeeding”* (P1, Exclusive breastfeeding mother).  *the weight was important to me* *because if she is small and heavy then it means she is healthy* (P10, Exclusive breastfeeding mother) |
|  |  | Birth size |  | ***“****I don’t know anything about the weight. I was influenced by the birth size to practice exclusive breastfeeding*  (P8, Exclusive breastfeeding mother)  ***“****The size will help me to see how big he or she is. I would want to maintain it and don't want her to be falling sick. I won’t introduce any other thing. I will maintain it or increase it”* (P3, Exclusive breastfeeding mother)  ***“****the size motivated me to give him only breast milk”* (P5, Exclusive breastfeeding mother). |
|  | **Other factors that influence the practice of exclusive breastfeeding.** | Advertisement |  | *“I have seen an advertisement that says that if you give the baby only breast milk, it develops her brains. That is why I planned to give her only breast milk”* (P9, Exclusive breastfeeding mother) |
|  |  | Work of mother |  | *“Because of the nature of my work (trading), I think that’s a factor which made me do exclusive breastfeeding. For those in the government sector such as banks, if you want to practice exclusive breastfeeding, it would be quite difficult. Even if you pump breast milk and place it in the fridge for the child, I don’t think it would be healthy for the child. The nature of my work also helps me practice exclusive breastfeeding, because my child is with me always and I can take her everywhere, I go”* (P10, Exclusive breastfeeding mother). |
|  |  | Enough breast milk |  | *“You can easily access breast milk when the baby is crying, it is available, you don’t prepare or have to go to the kitchen. It is available and brings love. Most mothers based on this to practice exclusive breastfeeding”* (Midwife).  *“I have a lot of breast milk so I do not see why I should add any other thing when the breast milk is there. I always give and she is always satisfied after taking it. So, I don’t have to add anything”* (P3, Exclusive breastfeeding mother). |
|  |  | Health workers advocacy |  | *“Yes, they taught us how to breastfeed. They will sometimes bring a doll and teach us how we should hold the breast, how you should feed the baby, the kind of food you can give it and how to keep yourself neat.”* (P7, Exclusive breastfeeding mother).  *“We normally explain exclusive breastfeeding to them for their understanding. If they understand the benefits of breastfeeding, then there is a need for them to start”* (Midwife). |
|  |  | Support from partner and other family members | *Partner*  *Mothers-in-law and friends* | *“Yes, my husband understands so I am okay”* (P6, Exclusive breastfeeding mother)  *“Left to him alone he says I should even do EBF for one year and I told him is not done like that”* (P10, Exclusive breastfeeding mother)  *“Some told me to give him food and others told me not to, but I had decided to practice exclusive breastfeeding. When they ask me to give the baby food, I only said yes knowing that I would not do it”* (P10, Exclusive breastfeeding mother).  *“They told me he will not have an appetite for food when he grows up and I told them that I will start giving him beans, maize and rice when he is six months. I believe he will have an appetite for food when he turns 6 months”* (P4, Exclusive breastfeeding mother). |
|  |  |  |  |  |
|  | **The benefit of exclusive breastfeeding** | Economic benefits |  | *“I should say that for the child and the mother, it saves money for her. It’s costless for the mother to buy breastmilk for the child. The father becomes happy because he is not spending much money on buying those things”* (Community Health Nurse).  *“I don’t buy feed for the baby, so I use that money to sew clothes for the baby”* (P4 Exclusive breastfeeding mother) |
|  |  | Family planning |  | *“When you breast the baby and you don’t stop breastfeeding the baby, in time, it becomes a family planning method for the mother”* (P7 Exclusive breastfeeding mother).  *“To the mother when you are doing exclusive breastfeeding, it prevents you from having an early pregnancy because you are breastfeeding on demand. It helps you not to get pregnant which is physiology by itself”* (Midwife). |
|  |  | Health benefits |  | *“It makes the child healthy and she doesn’t get diarrhoea. When you are doing exclusive breastfeeding all the nutrients that she needs are in the breast milk, so you don’t need to add any other thing”* (P3 Exclusive breastfeeding mother)    *“They said the exclusive breastfeeding protects them from diseases and it makes them grow healthy”* (P3 Non-exclusive breastfeeding mother) |
|  |  |  |  |  |
|  |  |  |  |  |
